# Idiopathic Systemic Capillary Leak Syndrome: A Clinical Case

**DOI:** 10.7759/cureus.50301

**Published:** 2023-12-10

**Authors:** Rui Soares Correia, Diana Pinho dos Santos, Marlene Delgado

**Affiliations:** 1 Internal Medicine, Centro Hospitalar Tondela-Viseu, Viseu, PRT; 2 Internal Medicine, Centro Hospitalar Universitário de São João, Porto, PRT

**Keywords:** hypovolemia, polycythemia, hemoconcentration, hypoalbuminemia, capillary hyperpermeability, clarkson’s disease, systemic capillary leak syndrome

## Abstract

Idiopathic systemic capillary leak syndrome (ISCLS) is a rare condition caused by the extravasation of intravascular fluids and proteins into the interstitial space due to increased vascular endothelium permeability. It is characterized by episodes of hypotension, hypoalbuminemia, and hemoconcentration with generalized edema. Its etiopathogenesis is unknown. However, it is associated with monoclonal gammopathy in more than 80% of cases. There is currently no targeted treatment, and the approach during a crisis is supportive, mainly to control blood pressure, maintain perfusion of vital organs, and prevent complications, such as acute pulmonary edema and organ failure due to ischemia, which are the primary causes of death.

We present the case of a 72-year-old man with generalized edema and pleural, pericardial, and peritoneal effusions whose laboratory results showed hypoalbuminemia, hypoproteinemia, and immunoglobulin G kappa monoclonal gammopathy. Other etiologies for severe hypoalbuminemia with anasarca were excluded after an exhaustive complementary study, leading to the diagnosis of ISCLS associated with monoclonal gammopathy. The patient showed progressive clinical improvement with albumin and diuretic therapy. However, they were readmitted to the hospital due to hypotension with multiorgan dysfunction and died a few hours later.

## Introduction

Clarkson et al. first described idiopathic systemic capillary leak syndrome (ISCLS), or Clarkson’s disease, in 1960. [1] Since then, approximately 260 cases have been reported in the literature worldwide [2].

ISCLS is a rare pathological condition characterized by acute episodes in which vascular endothelium dysfunction increases vascular permeability, resulting in extravasation of plasma and macromolecules (mainly proteins) into the interstitial compartment. Intravascular volume depletion causes hypotension, decreased tissue oxygenation, and even shock in severe cases. In addition, hemoconcentration occurs due to cell retention in the vessels, which may be responsible for thrombotic events [3,4]. Therefore, the crises of ISCLS are clinically characterized by the triad of hypotension, hypoalbuminemia, and hemoconcentration with generalized edema. They generally present in three phases: prodromal, capillary leakage, and recovery.

The first phase, also known as the prodromal phase, is characterized by nonspecific and mild symptoms that precede the leakage phase by 1-4 days. A history of respiratory infection or recent flu-like syndrome, including COVID-19, is common during this phase. The clinical triad develops during the capillary leakage phase, which has an average duration of four days (ranging from 1 to 27 days). Hypotension occurs in almost all patients and can be severe, leading to shock and tissue hypoperfusion. Hemoconcentration (average hematocrit of 60%) is an important diagnostic feature in ISCLS since it helps distinguish it from other causes of shock. Hypoalbuminemia is around 1.7 g/dL. This triad is accompanied by other clinical manifestations resulting from capillary leakage (generalized edema, pleural effusion, pericardial effusion, ascites, and even cerebral edema leading to encephalopathy) and systemic hypotension and hypoperfusion (cold skin, weak pulses, oliguria, agitation/obnubilation, and lactic acidosis). The transition to the recovery phase can occur suddenly. During this phase, extravasated fluids return to the intravascular space, leading to hemodynamic stability and edema regression through polyuria [5,6].

These episodes vary in severity and frequency and can be fatal. The primary complications of ISCLS are pulmonary edema (resulting from intensive fluid therapy), which can develop suddenly when patients enter the recovery phase, compartment syndrome (resulting from fluid extravasation into the muscular compartment), organ ischemia with consequent organ failure (the most common being acute kidney injury due to acute tubular necrosis, ischemic stroke, and ischemic hepatitis), and thrombotic events secondary to hemoconcentration (the most frequent being deep vein thrombosis, pulmonary embolism, and stroke). Acute pulmonary edema and organ failure are the primary causes of death in patients with ISCLS.

The complementary diagnostic approach is like any cause of shock (ISCLS is a combined form of distributive and hypovolemic shock). Given its frequent association with monoclonal gammopathy, additional tests such as serum protein electrophoresis and measurements of immunoglobulins and free light chains should be requested. The therapeutic approach to ISCLS is similar to that of septic shock. Hypotension requires immediate intervention to prevent complications of prolonged hypoperfusion, starting with intensive fluid therapy (preferably crystalloids, reserving colloids such as albumin for rescue therapy). Monitoring volume status is vital because inadequate resuscitation can lead to ischemic organ injury, while aggressive fluid therapy can cause pulmonary edema and compartment syndrome. The transition from the leakage to the recovery phase should be recognized by a decrease in intravenous fluid requirements to maintain adequate perfusion. Therefore, most patients require diuretics to prevent hypervolemia and its complications [7].

There are no specific pharmacological therapies for acute ISCLS episodes except for symptomatic support. However, some cases report the effectiveness of specific therapeutic modalities, including intravenous immunoglobulin and combined terbutaline with aminophylline. The latter have also shown efficacy as prophylaxis to prevent the recurrence of future crises. The five-year survival rate is around 70%. Some patients with monoclonal gammopathy develop multiple myeloma over time.

We present the case of a 72-year-old man with generalized edema and pleural, pericardial, and peritoneal effusions whose laboratory results showed hypoalbuminemia, hypoproteinemia, and immunoglobulin G kappa monoclonal gammopathy. Other etiologies for severe hypoalbuminemia with anasarca were excluded after an exhaustive complementary study, leading to the diagnosis of ISCLS associated with monoclonal gammopathy. The patient showed progressive clinical improvement with albumin and diuretic therapy. However, they were readmitted to the hospital due to hypotension with multiorgan dysfunction and died a few hours later.

## Case presentation

**Figure 1:**
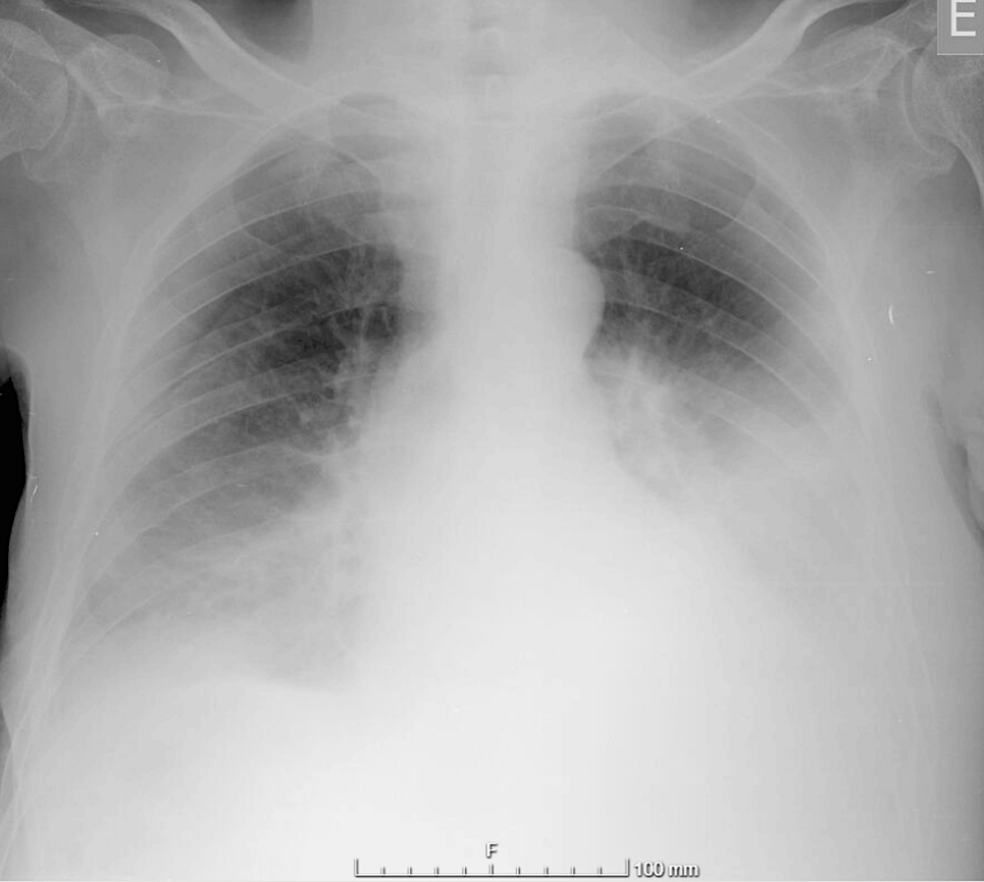
Admission chest X-ray with bilateral pleural effusion

A 72-year-old man with a history of arterial hypertension, type 2 diabetes mellitus, stage 3b chronic kidney disease, and chronic obstructive pulmonary disease. He was being treated with furosemide, perindopril, amlodipine, and saxagliptin. The patient presented to the emergency department with a two-week history of generalized edema, increased abdominal volume, and dyspnea on slight exertion. On physical examination, he was oriented, afebrile, eupneic, and had a blood pressure of 117/82 mmHg. He had decreased breath sounds at the bases of both hemithoraces and signs of ascites and edema in the eyelids, chest, abdominal wall, scrotum, and lower limbs. Blood gas analysis showed hypoxemic respiratory failure (arterial oxygen pressure (PaO_2_) of 54 mmHg and arterial oxygen saturation (SaO_2_) of 90%) without lactic acidosis. Blood samples showed normocytic and normochromic anemia (hemoglobin (Hb) of 10.2 g/dL and hematocrit (Ht) of 30.3%) with normal leukocytes and platelets, hypoproteinemia (4 g/dL), severe hypoalbuminemia (1.2 g/dL), stable renal function (creatinine of 2.1 mg/dL), and normal electrolytes. Additionally, inflammatory biomarkers, liver profile, and N-terminal-pro B-type natriuretic peptide were within normal limits. Chest X-ray showed bilateral pleural effusion (Figure 1), and abdominal ultrasound revealed moderate ascites without other ultrasound changes, including liver morphology.

He was admitted to the hospital for clinical stabilization and further investigation of the underlying condition.

Analytical studies (Table 1) showed normal iron kinetics, thyroid function, serum cortisol, and negative autoimmunity. Serum protein electrophoresis showed an increased gamma peak and immunofixation showed monoclonal IgG bands with kappa light chains. Urine analysis showed no proteinuria.

**Table 1:** Summary of laboratory results Hb: Hemoglobin; Ht: Hematocrit; AST: Aspartate aminotransferase; ALT: Alanine transaminase; NT-proBNP: N-terminal pro-B-type natriuretic peptide; CRP: C-reactive protein; TSH: Thyroid-stimulating hormone; T4L: T4 lysozyme

Test		First admission	Readmission	Reference range
Hb (g/dL)		10.2	9.4	14.0-18.0
Ht (%)		30.3	28.1	40.0-54.0
Albumin (g/dL)		1.2	1.5	3.5-5.0
Total serum protein (g/dL)		4.0	4.4	6.6-8.7
Creatinine (mg/dL)		2.1	3.3	0.6-1.3
AST (UI/L)		46.0	68.0	4.0-43.0
ALT (UI/L)		38.0	68.0	4.0-43.0
Total bilirubin (mg/dL)		0.2		0.2-1.1
Prothrombin (%)		75.0	49.0	70.0-100.0
Troponin I (ng/L)			3285.4	37.5-80.4
NT-proBNP (pg/mL)		7844.0	16382.0	0.0-125.0
CRP (mg/dL)		1.2	0.7	0.0-0.5
TSH (mUI/L)		3.5		0.6-4.8
T4L (ng/dL)		1.1		0.9-1.8
Serum cortisol (ug/dL)		13.4		5.3-22.5
Serum immunoglobulin levels	IgA (mg/dL)	496.0		40.0-350.0
	IgG (mg/dL)	1686.0		650.0-1600.0
	IgM (mg/dL)	87.0		50.0-300.0
Blood serum light chains	Lambda mg/dL	763.0		280.0-665.0
	Kappa (mg/dL)	1470.0		598.0-1329.0

**Figure 2:**
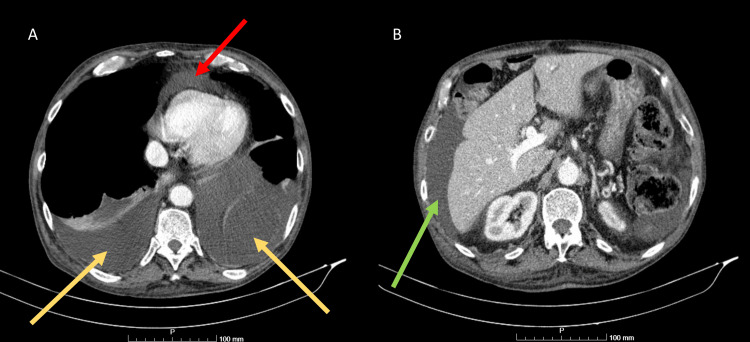
CT-scan A: Thoracic CT-scan: pericardial effusion (red arrow) and pleural effusion (yellow arrows); B: Abdominal CT-scan: ascites (green arrow)

The transthoracic echocardiogram showed a small pericardial effusion without signs of tamponade, with normal heart cavities, absence of valvular heart disease, and normal biventricular systolic function. A thoraco-abdominopelvic computerized tomography (CT) scan showed a large symmetrical bilateral pleural effusion, small pericardial effusion, moderate ascites, diffuse subcutaneous fat densification indicating edema, and segmental pulmonary thromboembolism (Figure 2).

The pleural fluid study was compatible with transudate, amicrobial, with no neoplastic cells and normal immunophenotyping. The endoscopic study of the digestive tract revealed no changes. Fecal α1-antitrypsin was normal. The medulogram with immunophenotyping was normal. However, the bone biopsy showed less than 10% of plasma cells, compatible with monoclonal gammopathy of undetermined significance (MGUS).

After excluding other causes of anasarca with severe hypoalbuminemia and monoclonal IgG-kappa light chain gammopathy, the ISCLS diagnosis was considered.

**Figure 3:**
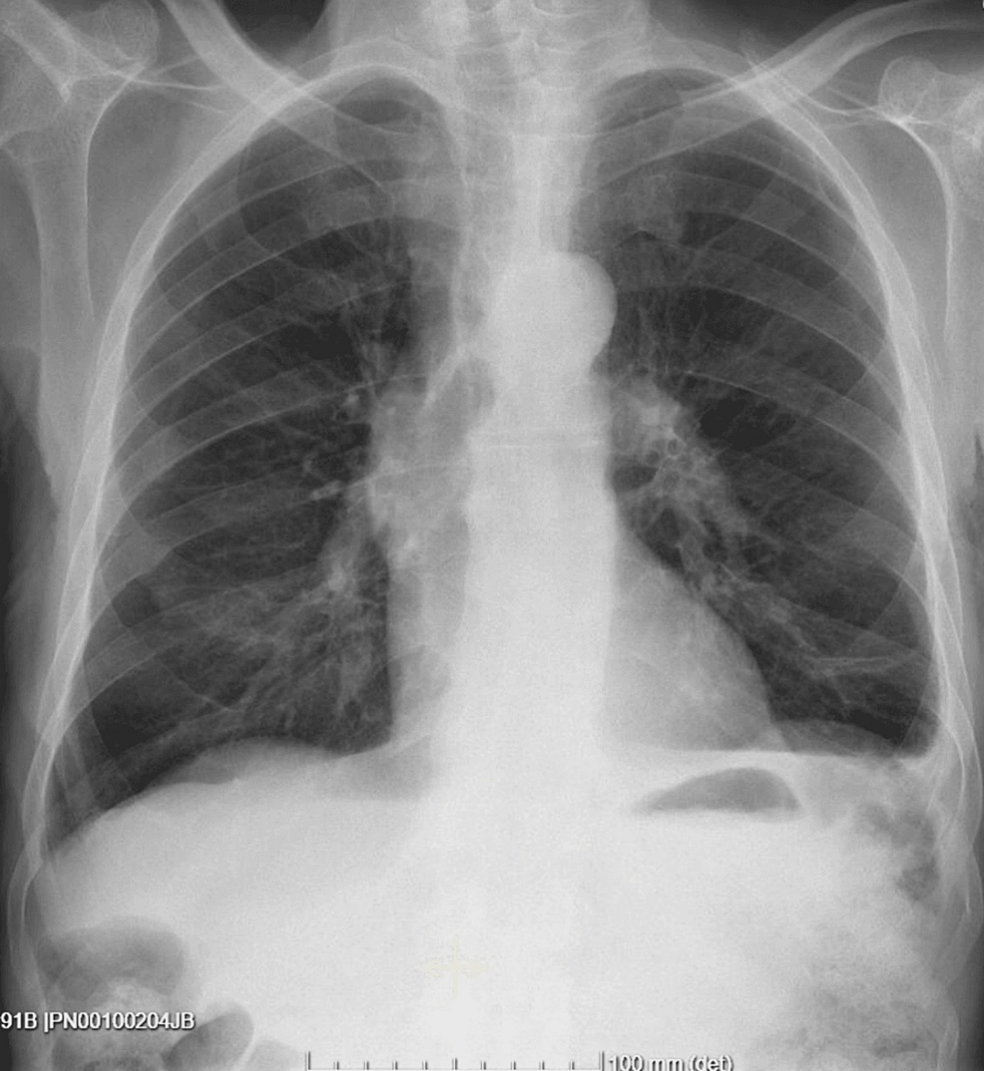
Chest X-ray at the time of discharge

The patient underwent therapy with albumin and loop diuretics and showed favorable clinical evolution with generalized edema regression and significantly decreased pleural (Figure 3) and ascitic effusions (with the resolution of hypoxemia) with increased in albuminemia (2.0 mg/dL) and improved renal function (creatinine of 1.6 mg/dl). 

**Figure 4:**
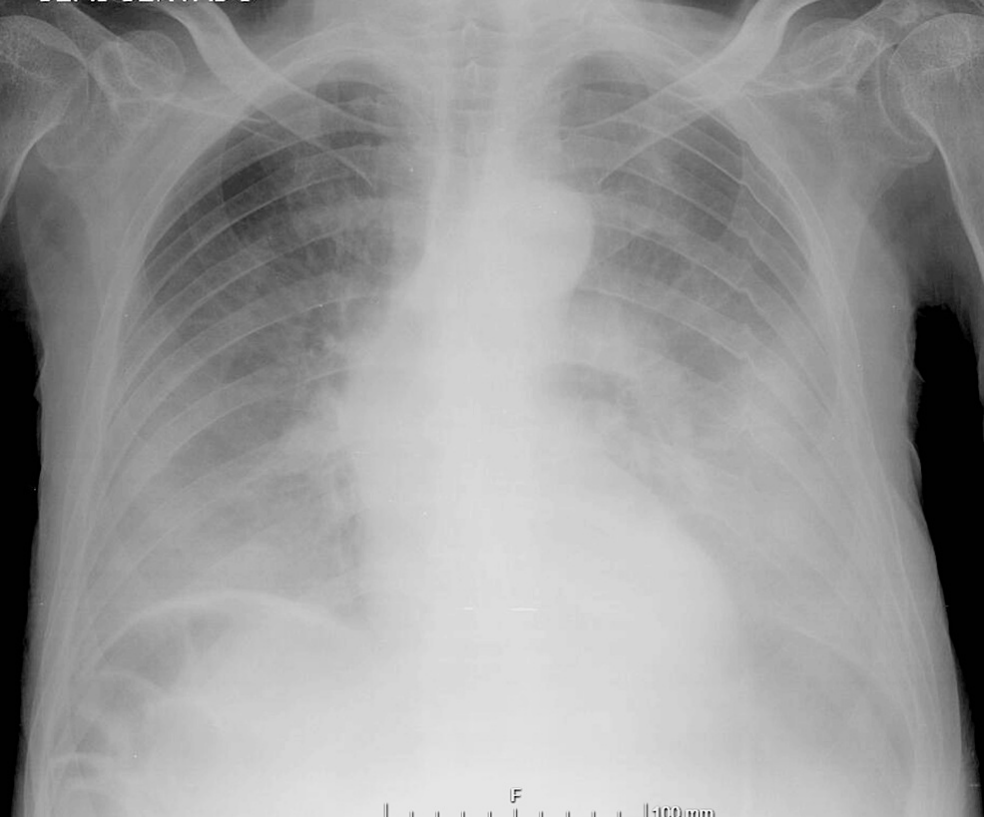
Chest X-ray on readmission

A month and a half after hospital discharge, the patient was readmitted to the hospital with a two-day history of obtundation, aphasia, inability to walk, and vomiting. On observation, he was alert but non-cooperative, with roving eye movements and orthophoria. There was no verbal response and no evident focal neurological deficits. He was hypotensive (98/56 mmHg) and was eupneic but had decreased vesicular sounds at both bases of the hemithoraces and no peripheric edema. Blood gas analysis showed compensated lactic metabolic acidosis with a pH of 7.45, partial pressure of carbon dioxide (pCO_2_) of 16 mmHg, bicarbonate (HCO_3_) of 11 mmol/L, and lactates of 2.7 mmol/L. Blood analysis revealed anemia (Hb of 9g/dL and Ht of 28%), hypoalbuminemia of 1.5mg/dL, elevated troponin I of 3285 ng/mL, acute kidney injury (creatinine of 4.0 mg/dL and urea of 229 mg/dL), and mild transaminase elevation (aspartate aminotransferase of 78 UI/L and alanine transaminase of 63 UI/L). A chest X-ray showed signs of pulmonary congestion and bilateral pleural effusion (Figure 4). The electrocardiogram showed sinus tachycardia without signs of acute ischemia. A cranial CT scan revealed no acute brain injuries.

Despite implementing therapeutic measures, including diuretics, the patient died a few hours after admission due to respiratory failure.

## Discussion

Since the diagnosis of ISCLS is made by exclusion, additional studies aim to exclude causes of capillary leakage, both due to increased hydrostatic pressure in the capillaries (such as heart failure, renal failure, and cirrhosis), decreased oncotic pressure (such as nephrotic syndrome, protein-losing enteropathy, and liver failure), or increased capillary permeability (such as sepsis, systemic inflammatory response syndrome, certain infections, acute pancreatitis, anaphylaxis, and drug reactions). In the presented case, this study was performed, concluding that there were no identifiable causes to explain the severe anasarca resulting from severe hypoalbuminemia.

While there was no evidence of arterial hypotension during the first hospitalization episode, it can be assumed that given the patient’s history of hypertension, the presented blood pressure values correspond to relative hypotension, especially because blood pressure values increased to mild hypertension levels during clinical improvement. However, given the duration of the condition, it is likely that the patient was in the final stage of the extravasation phase or transitioning to the recovery phase, which may explain his hemodynamic stability and lack of systemic hypoperfusion signs.

The absence of hemoconcentration could potentially be explained by the fact that the patient had chronic anemia due to chronic kidney disease, which could contribute to masking his already low Hb and Ht levels. The documentation of pulmonary embolism in the thoracic CT scan might indicate hemoconcentration.

The second hospitalization episode demonstrates the cyclic nature of this condition and manifested as hypotension with tissue hypoperfusion and ischemic organ injury (myocardial injury and renal injury), vascular leakage manifestations with pulmonary edema and pleural effusion, and encephalopathy, the latter due to cerebral hypoperfusion and/or cerebral edema.

The presence of MGUS in this case supports the ISCLS diagnosis.

## Conclusions

ISCLS is a rare and underdiagnosed condition due to the nonspecific nature of its clinical manifestations, its rapid progression with severe complications, and its high mortality rates, highlighting the importance of considering this condition in the differential diagnosis of a patient with anasarca and hypoalbuminemia.

The medical community should be aware of this condition since early diagnosis and timely treatment improve prognosis and allow for further development of targeted therapy in the future.
